# Supplementary material for: Combination Treatment to Improve Mucociliary Transport of Pseudomonas aeruginosa Biofilms
Source: bioRxiv. 2023 Aug 14:2023.08.14.553173. Preprint. [Version 1] doi: 10.1101/2023.08.14.553173 (PMC10461968; doi:10.1101/2023.08.14.553173)
Supplement: Supplement 1 [file NIHPP2023.08.14.553173v1-supplement-1.pdf]

bioRxiv preprint doi: https://doi.org/10.1101/2023.08.14.553173; this version posted August 14, 2023. The copyright holder for this preprint (which was not certified by peer review) is the author/funder, who has granted bioRxiv a license to display the preprint in perpetuity. It is made available under aCC-BY 4.0 International license.

**Supplemental Figures**

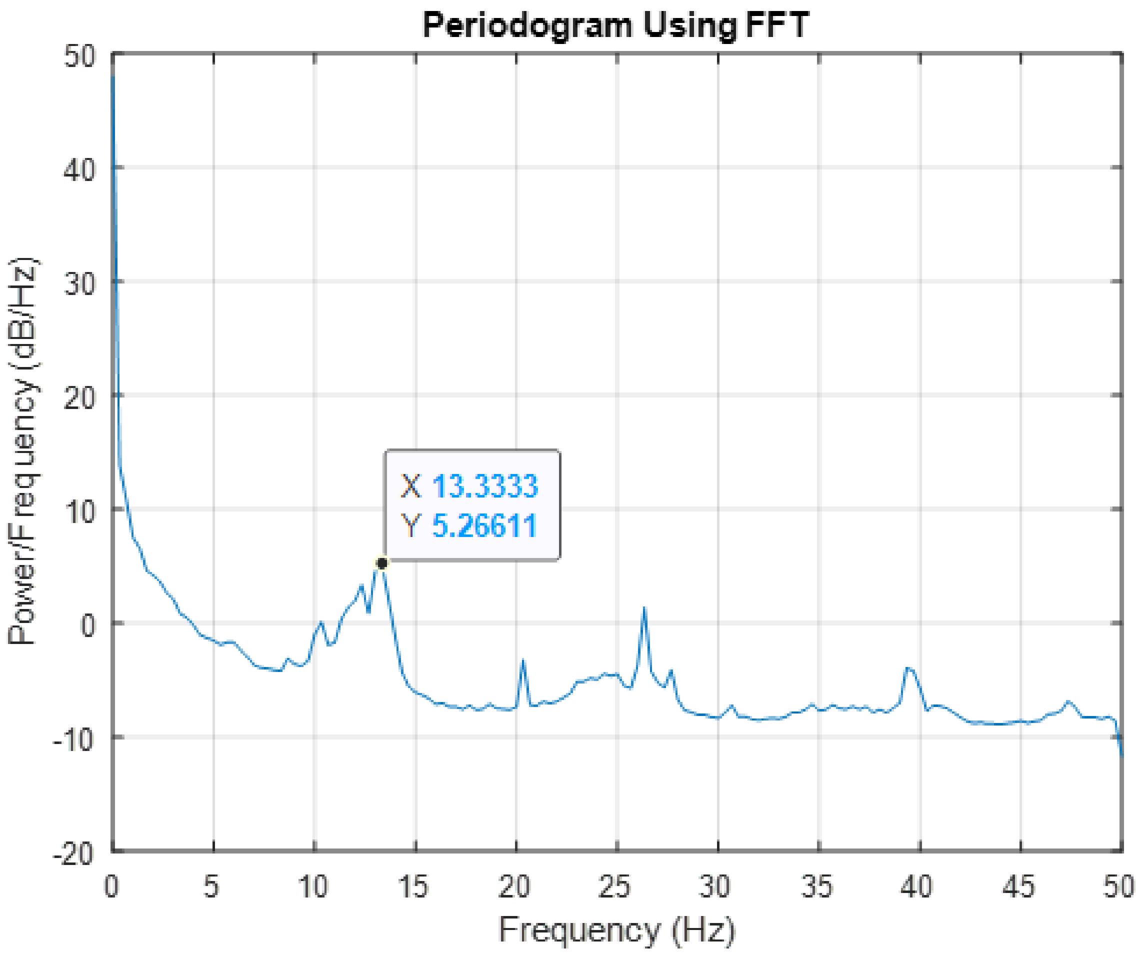


Supplemental Figure 1. Representative periodogram of cilia beat frequency quantification.

bioRxiv preprint doi: https://doi.org/10.1101/2023.08.14.553173; this version posted August 14, 2023. The copyright holder for this preprint (which was not certified by peer review) is the author/funder, who has granted bioRxiv a license to display the preprint in perpetuity. It is made available under aCC-BY 4.0 International license.

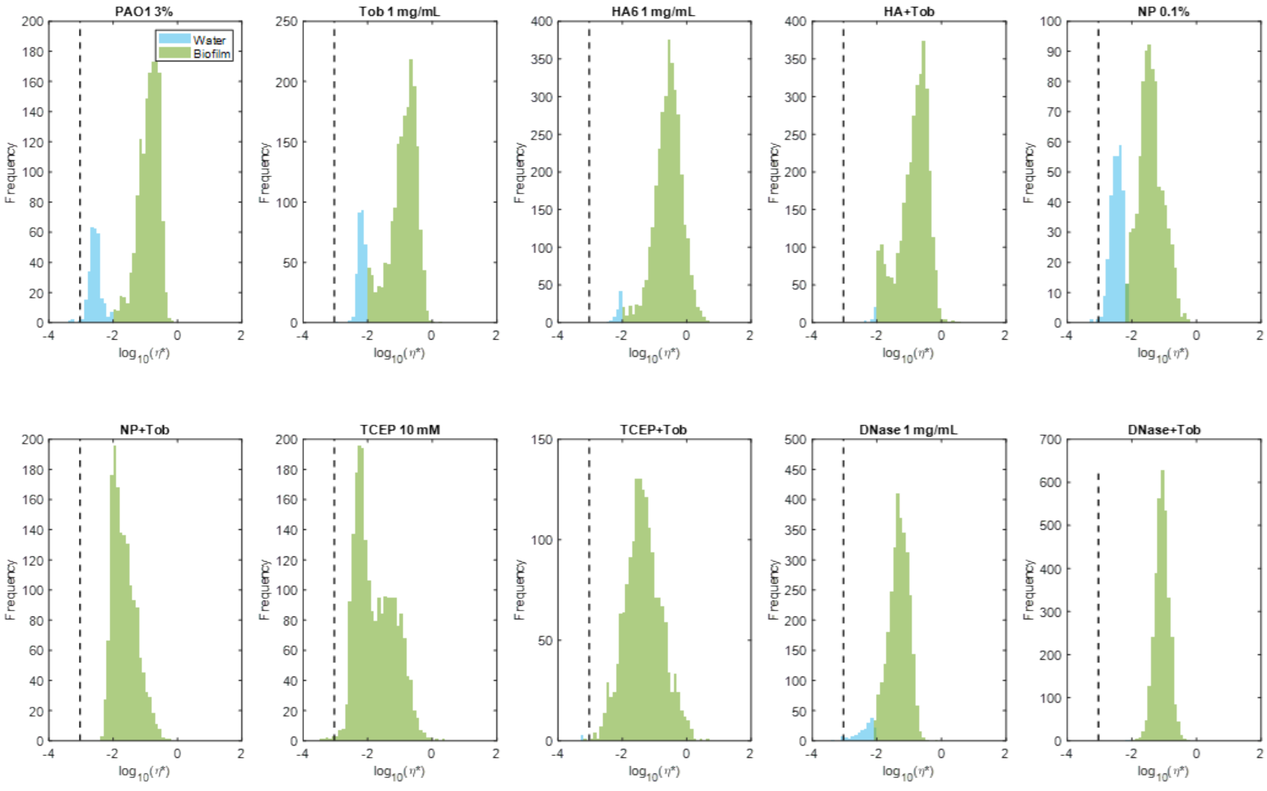


Supplemental Figure 2. Complex viscosity distribution of treated PAO1 biofilms. Gaussian mixture modeling was used to separate the watery component (blue) from the more solid-like biofilm component (green).

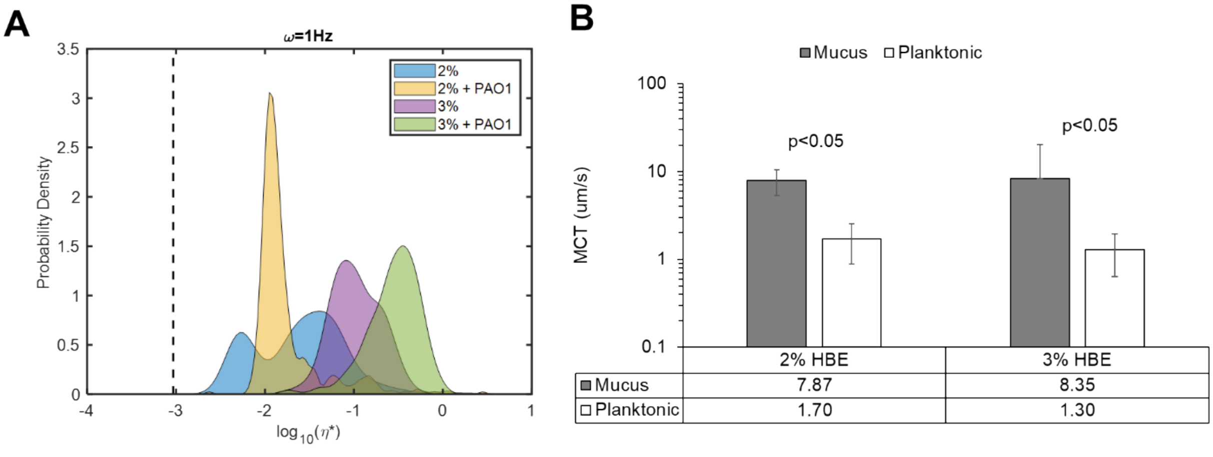


Supplemental Figure 3. Effect of planktonic bacteria on A) mucus rheology and B) transport. Statistical significance was determined using single factor ANOVA with post hoc Tukey Kramer analysis.

bioRxiv preprint doi: https://doi.org/10.1101/2023.08.14.553173; this version posted August 14, 2023. The copyright holder for this preprint (which was not certified by peer review) is the author/funder, who has granted bioRxiv a license to display the preprint in perpetuity. It is made available under aCC-BY 4.0 International license.

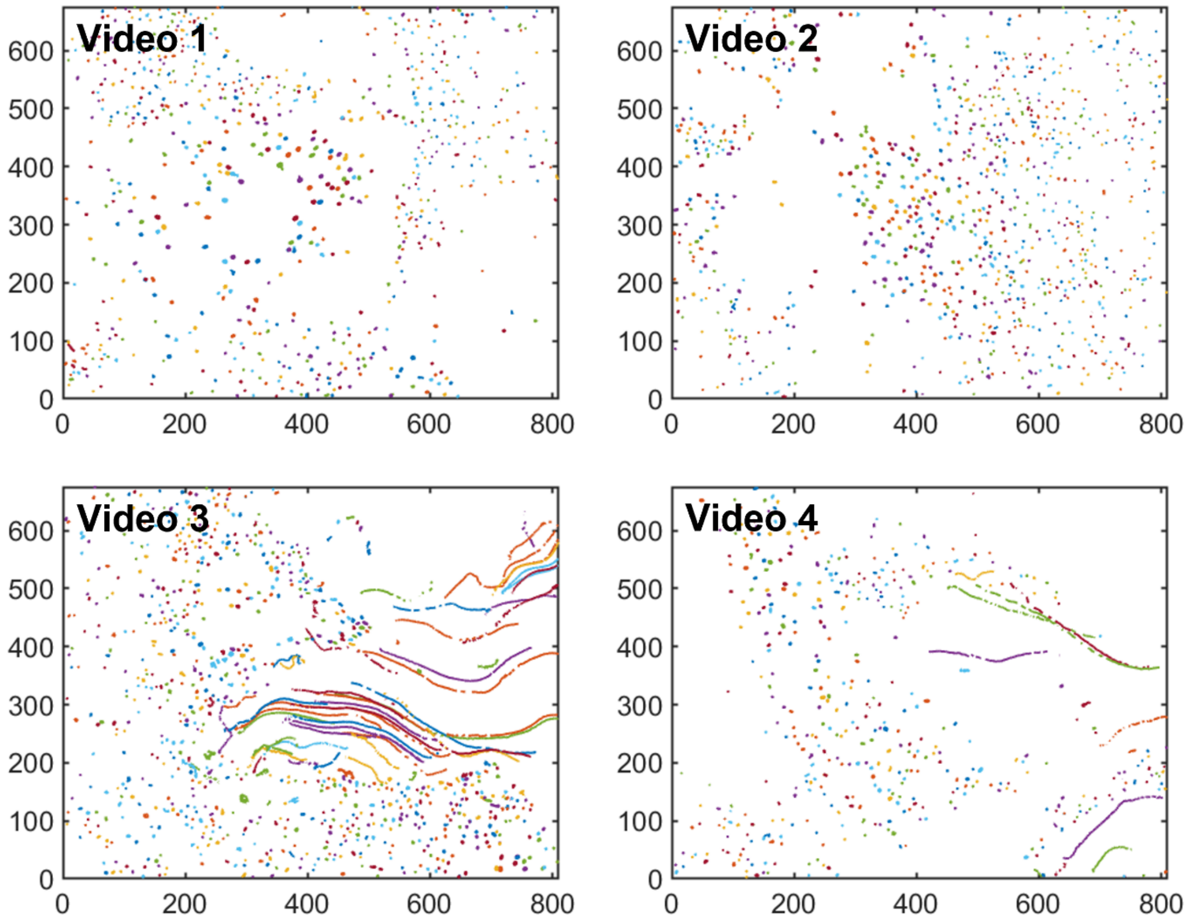


Supplemental Figure 4. Representative images of biofilm mucociliary transport after treatment with 10 mM TCEP and 1 mg/mL Tob

bioRxiv preprint doi: https://doi.org/10.1101/2023.08.14.553173; this version posted August 14, 2023. The copyright holder for this preprint (which was not certified by peer review) is the author/funder, who has granted bioRxiv a license to display the preprint in perpetuity. It is made available under aCC-BY 4.0 International license.

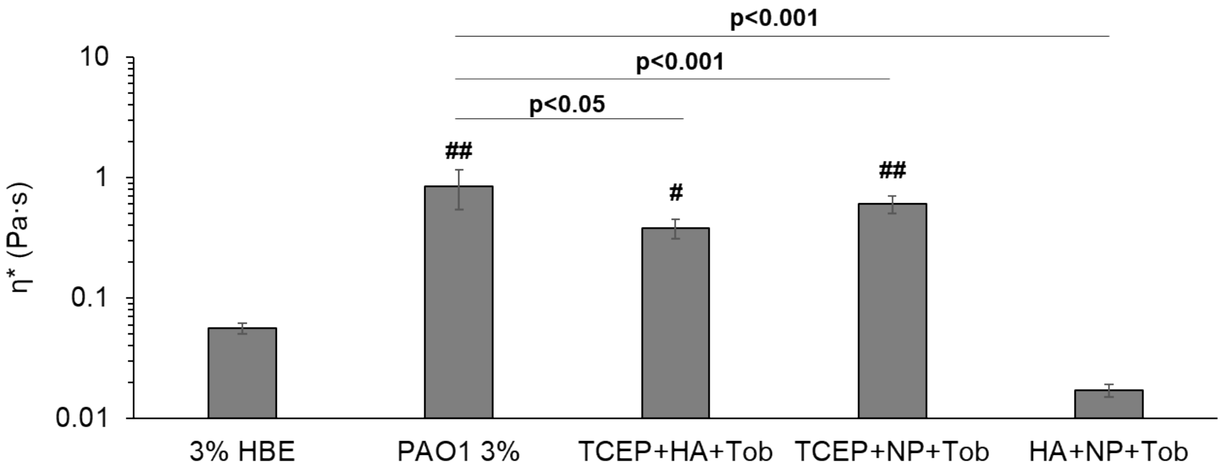


**# p<0.01, ## p<0.001 compared to HBE**

Supplemental Figure 5. Macrorheology of triple combination treated biofilms. Data is representative of the average ± standard deviation of three separately treated and evaluated samples. Statistical significance was determined with single factor ANOVA with post hoc Tukey analysis.

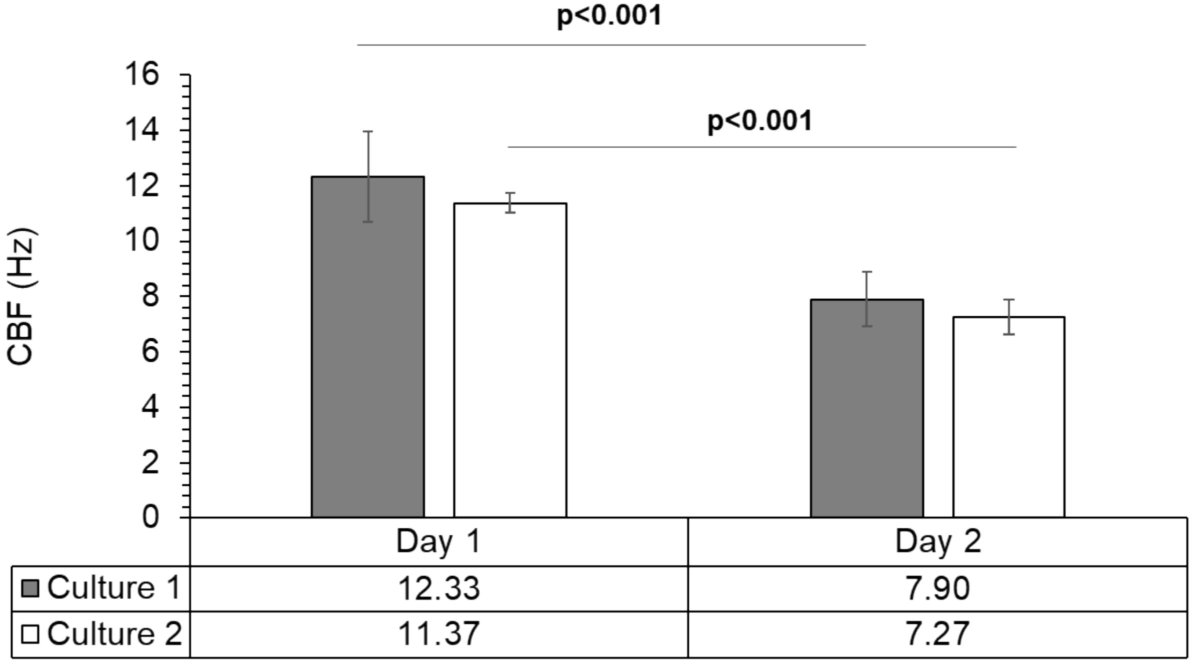


Supplemental Figure 6. Cilia beat frequency of two racetrack cultures as a function of time post infection with a PAO1 biofilms. Data is representative of the average ± standard deviation of six separate videos for each culture. Statistical significance was determined with single factor ANOVA with post hoc Tukey analysis.
